# A validation study comparing existing prediction models of acute kidney injury in patients with acute heart failure

**DOI:** 10.1038/s41598-021-90756-9

**Published:** 2021-05-27

**Authors:** Tao Han Lee, Pei-Chun Fan, Jia-Jin Chen, Victor Chien‐Chia Wu, Cheng-Chia Lee, Chieh-Li Yen, George Kuo, Hsiang-Hao Hsu, Ya-Chung Tian, Chih-Hsiang Chang

**Affiliations:** 1 Kidney Research Center, Department of Nephrology, Chang Gung Memorial Hospital, Linkou branch, No. 5, Fuxing Street, Guishan Dist., Taoyuan City, 33305 Taiwan ROC; 2 Graduate Institute of Clinical Medical Science, College of Medicine, Chang Gung University, Taoyuan, Taiwan ROC; 3 Division of Cardiology, Chang Gung Memorial Hospital, Linkou Medical Center, Taoyuan, Taiwan ROC

**Keywords:** Heart failure, Heart failure, Acute kidney injury, Risk factors

## Abstract

Acute kidney injury (AKI) is a common complication in acute heart failure (AHF) and is associated with prolonged hospitalization and increased mortality. The aim of this study was to externally validate existing prediction models of AKI in patients with AHF. Data for 10,364 patients hospitalized for acute heart failure between 2008 and 2018 were extracted from the Chang Gung Research Database and analysed. The primary outcome of interest was AKI, defined according to the KDIGO definition. The area under the receiver operating characteristic (AUC) curve was used to assess the discrimination performance of each prediction model. Five existing prediction models were externally validated, and the Forman risk score and the prediction model reported by Wang et al. showed the most favourable discrimination and calibration performance. The Forman risk score had AUCs for discriminating AKI, AKI stage 3, and dialysis within 7 days of 0.696, 0.829, and 0.817, respectively. The Wang et al. model had AUCs for discriminating AKI, AKI stage 3, and dialysis within 7 days of 0.73, 0.858, and 0.845, respectively. The Forman risk score and the Wang et al. prediction model are simple and accurate tools for predicting AKI in patients with AHF.

## Introduction

Acute heart failure (AHF) with abrupt onset dyspnoea, sensation of suffocation, and sometimes, pink frothy expectoration is a leading cause of hospitalization. Acute kidney injury (AKI) or worsening renal function (WRF), the term used in some previous studies, are common complications among patients with AHF, with an incidence of 21–45%^1–3^. Previous studies have revealed that the development of AKI in patients with AHF results in longer hospital stays, higher readmission rates, and increased short- and long-term mortality^4–7^. Smith et al. further reported that even a slightly increased creatinine level (≥ 0.2 mg/dL) increases the risk of mortality among patients with AHF^8^. In the past few years, investigators have reported that AHF patients might experience “congestion”, named to describe signs and symptoms of extracellular fluid accumulation that results in increased cardiac filling pressure, and “renal congestion” has been recognized as part of systemic congestion. Renal congestion, resulting from lower cardiac output, tubuloglomerular feedback, increased intra-abdominal pressure and increased venous pressure, has been viewed as a contributor to renal function impairment in AHF^9^. Nevertheless, evidence has indicated that in those patients decongested at discharge, in-hospital WRF was not associated with worse outcomes^10,11^. However, there is no reliable clinical or laboratory marker to distinguish WRF caused by renal congestion and “true” acute kidney injury^9,11,12^. Therefore, the early recognition and identification of patients who are at high risk of developing AKI is still essential for early prevention and treatment in patients with AHF.

For these reasons, many studies have focused on identifying relevant risk factors, with some having derived AKI prediction models in patients with AHF^1,13–17^. The Forman risk score, first reported in 2004, was initially based on hospitalized heart failure patients but was later externally validated in AHF patients, and it is arguably the best-known prediction model worldwide^13^. Following the subsequently developed Basel risk score, prediction models were also proposed by Wang et al. and Zhou et al. between 2011 and 2016^1,15,16^. However, the definition of AKI or WRF varies in these studies due to the AKI classification changing from the RIFLE (Risk, Injury, Failure, Loss of kidney function, and End-stage kidney disease) classification and AKIN (Acute Kidney Injury Network) criteria to the KDIGO (Kidney Disease Improving Global Outcome) guidelines in the past few years^18–20^. In addition to the changing AKI definition and classification by year, these existing prediction models also vary by population, region, sample size and research methods. Considering the importance of early identification, prevention, and intervention of AKI in patients with AHF, revalidating the performance and discrimination of these prediction models together and according to the current AKI definition seems to be necessary. Therefore, we aim to externally validate the existing prediction models for AKI in patients with AHF based on the KDIGO Clinical Practice Guidelines for Acute Kidney Injury.

## Methods

### Data source

This study was based on the electronic medical records of the Chang Gung Research Database (CGRD) from the Chang Gung Medical Foundation. The database incorporates data from the nationwide Chang Gung Memorial Hospital system, which is the largest health care system of its kind in Taiwan, comprising two medical centres, two regional hospitals, and three district hospitals. The CGRD consists of clinical epidemiological data, laboratory data, inpatient and outpatient records, emergency medical records, pathology reports, and disease category data. The overall coverage rates of the CGRD are approximately 20% for outpatients and 12% for inpatients for the entire Taiwanese population. More detailed information about the CGRD has been reported in previous studies^21,22^. Its disease diagnoses are coded using the International Classification of Diseases, Ninth Revision, Clinical Modification (ICD-9-CM) for records before 2016 and the International Classification of Diseases, Tenth Revision, Clinical Modification (ICD-10-CM) for those thereafter. The Institutional Review Board of Chang Gung Memorial Hospital approved the study (approval number: CGMHIRB No. 202000915B0) and waived the need for informed consent due to the retrospective nature of the study, which did not compromise the privacy of any patients. In this study, all the methods were performed in accordance with the Declaration of Helsinki.

### Study population

We analysed the records of patients who had emergency department visits and were subsequently admitted due to acute heart failure (ICD-9-CM diagnostic code: 428; ICD-10-CM diagnostic code: I50) between January 1, 2008, and December 31, 2018, at all 7 Chang Gung Memorial Hospital branches located in Linkou, Taipei, Taoyuan, Keelung, Yunlin, Chiayi, and Kaohsiung, which span northern to southern Taiwan. When a patient had multiple AHF episodes, the first episode of AHF hospitalization between 2008 and 2018 was selected as the index hospitalization. The admission date of the index AHF hospitalization was used as the index date. The first record of laboratory examination results was used as the baseline laboratory data; the first records of vital sign data and medication treatment within 48 h after admission were also collected for further analysis.

**Figure 1:**
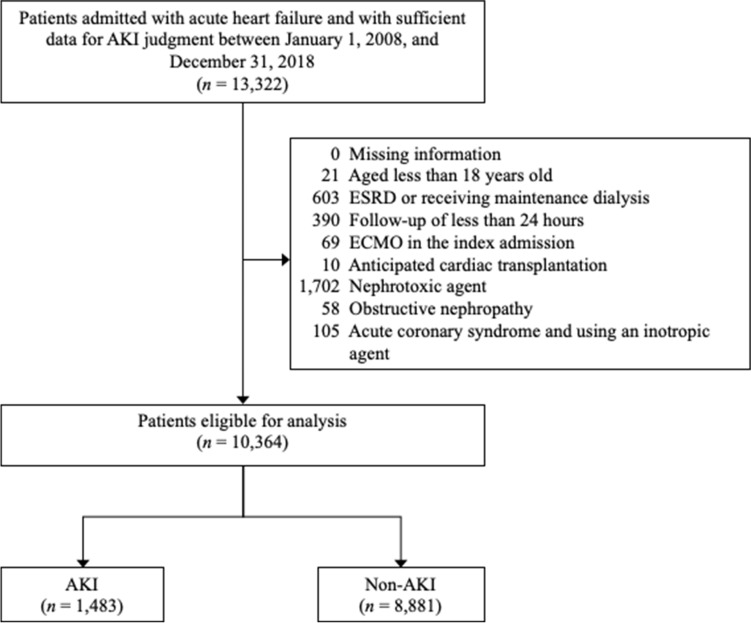
Flow chart for patient selection.

Patients without sufficient data for AKI assessment were not included, such as those without baseline creatinine data or a second creatinine examination within 7 days of admission. The remaining patients were excluded if they met any of the following criteria: (1) were younger than 18 years old, (2) had end-stage renal disease or were undergoing maintenance dialysis, (3) had follow-up of less than 24 h, (4) received extracorporeal membrane oxygenation during the index admission, (5) anticipated cardiac transplantation, (6) received a nephrotoxic agent (including contrast agents, nonsteroidal anti-inflammatory drugs, aminoglycoside, and vancomycin) within 4 weeks of admission or during the index admission, (7) had obstructive nephropathy, and (8) had acute coronary syndrome with inotropic agents used during the index admission. After exclusion, 10,364 patients remained eligible for the study, of whom 1483 (14.3%) had AKI (Fig. 1).

### Existing prediction models and covariates

A review of previous studies found five prediction models or risk factor studies for AKI prediction in patients with AHF. All five were externally validated in this study.

The prediction models were as follows: the Forman risk score, reported in 2004 and based on four prediction factors, including underlying diseases as well as clinical and laboratory parameters^13^; the Basel risk score, reported in 2011 and using chronic kidney disease, bicarbonate level, and outpatient diuretic treatment as indices^1^; the prediction model reported by Wang et al. in 2013 based on a data analysis of 1709 patients and using 8 prediction factors^15^; the prediction model reported by Zhou et al. in 2016 that combines clinical parameters and novel urine biomarkers for AKI prediction in AHF patients^16^, though we were unable to include the NT-proBNP and urine biomarkers used by Zhou et al. because the CGRD did not include these data; and the study by Verdiani et al. in 2010 investigating predication of AKI in hospitalized AHF patients^14^.

The study populations, publication years, heart failure criteria, and full lists of predictors of the prediction models are summarized in Table 1.

**Table 1:** Existing prediction models for acute kidney injury in patients with acute heart failure.

Prediction model	Prediction factors	Population and patient number	Study years	Heart failure criteria	Specificity and sensitivity	AUC
2004 Forman risk score^13^	CHF, DM, SBP level, creatinine	1004 patients, U.S	1997–1998	ICD-9-CM†	Sen 81%; Spe 62%	AUC 0.65*
2010 Verdiani^14^	Age, CKD, heart rate, creatinine, CCB use, digoxin use	394 patients, Italy	2002–2008	Standard Framingham criteria	-	-
2011 Basel risk score^1^	CKD, bicarbonate outpatient, diuretics	575 patients, Switzerland	2001–2002, 2006–2010	2008 ESC guidelines	-	AUC 0.71 (95%CI 0.63–0.79)
2013 Wang^15^	Age, heart functional class, admission times for acute heart failure, SBP level, creatinine, sodium, proteinuria, IV furosemide use	1709 patients, China	2004–2011	ICD-10-CM‡, 2012 ESC guidelines	Sen 70.0%; Spe 70.6%	AUC 0.76 (95% CI: 0.73–0.79)
2016 Zhou^16^	Age, sex, CKD, albumin, NT-proBNP, uNGAL, uAGT	507 patients, China	2011–2014	2005 ESC guidelines	-	AUC 0.765 for clinical model alone AUC 0.874 for prediction model

*AUC* area under the receiver operating characteristic curve; *CCB* calcium channel blocker; *CHF* congestive heart failure; *CKD* chronic kidney disease; *DM* diabetes mellitus; *ESC* European Society of Cardiology; *ICD-9-CM* International Classification of Diseases, *ICD-10-CM* International Classification of Diseases; tenth Revision, Clinical Modification; IV: intravenous therapy; *NT-proBNP* N-terminal pro-brain natriuretic peptide; *SBP* systolic blood pressure; *uAGT* urinary angiotensinogen; *uNGAL* urinary neutrophil gelatinase-associated lipocalin; *U.S.* United State.

*AUC is not calculated in original article. AUC 0.65 was documented by 2011 Basel risk score and 2013 Wang study.

^†^Heart failure was identified using ICD-9-CM codes 428.0, 428.1, 402.01, 402.11, 402.91, 404.01, 404.03, 404.11, 404.13, 404.91, and 404.93.

^‡^Heart failure was identified using ICD-10-CM codes I50.102, I50.106, I50.107, I50.902, I50.903, I50.904, I50.908, I50.910, and I50.911.

### Outcome definition

The primary outcome was the development of AKI within 7 days after admission. The first record of the creatinine level during emergency department admission was used as the baseline creatinine level, and AKI was defined as an increase in serum creatinine by 0.3 mg/dL within 48 h or a 50% increase in serum creatinine within 7 days, in accordance with the KDIGO Clinical Practice Guidelines for Acute Kidney Injury^20^. This study also validated the performance of existing prediction models in predicting serious AKI events, including AKI stage 3 and dialysis. Stage 3 AKI was defined as a ≥ 200% increase in serum creatinine, a serum creatinine concentration of ≥ 4 mg/dl, or the initiation of dialysis within 7 days of study enrolment, according to the KDIGO guidelines. Urine output was not used to define AKI because these data were not complete in the CGRD.

To identify the clinical end point of acute kidney injury, we also evaluated the development of major adverse kidney events (MAKEs) on or after the 8^th^ day following the index date. MAKEs were defined as the composite of chronic kidney disease (an estimated glomerular filtration rate [eGFR] decline of > 25% from baseline), end-stage renal disease (ESRD) requiring chronic renal replacement therapy, and all-cause mortality^23,24^. We assessed MAKEs within 1 year of AKI diagnosis and from the index date to the final visit date, the date of death, the date of event occurrence, or December 31, 2018, whichever came first. Only patients with a follow-up duration of > 7 days were included in the MAKE analysis.

### Statistical analysis

Due to the presence of a substantial amount of missing data, we imputed the data using the single expectation maximization (EM) method for the primary analysis in this study. To test the robustness of the results, only patients with complete data were retained and used in the sensitivity analysis. It was noted that the missing rate of bicarbonate data was particularly high (59%).

The characteristics of the patients in the AKI and non-AKI groups were compared using the Mann–Whitney U-test for continuous variables (due to the lack of normality) and the chi-square test for categorical variables. The discrimination ability of individual scores in predicting an outcome of interest (i.e., AKI, AKI stage 3, or dialysis) in patients with AHF was determined using the area under the receiver operating characteristic curve (AUC). Optimal cut-off points were determined using the Youden index, and the corresponding sensitivity and specificity were calculated. The AUCs among the existing prediction models were compared in a pairwise manner using the DeLong test. In addition, the calibration performance of each score was assessed using the Hosmer–Lemeshow (HL) goodness-of-fit test, with smaller statistics (chi-square) indicating a smaller discrepancy between the predicted probability and observed AKI event for the prediction models. The patients were divided into two subgroups according to the optimal cut-off of each score. The risk of MAKEs on or after the 8^th^ day following the index date was compared between the higher and lower cut-offs of each score using the Cox proportional hazards model.

All tests were 2-tailed, and *P* < 0.05 was considered statistically significant. Data analyses were conducted using SPSS 25 (IBM SPSS Inc., Chicago, Illinois).

### Ethics approval and consent to participate

The Institutional Review Board of Chang Gung Memorial Hospital approved the study (approval number: CGMHIRB No. 202000915B0) and waived the need for informed consent due to the retrospective nature of the study, which did not compromise the privacy of any patients.

## Results

### Baseline characteristics

The patients’ characteristics at baseline are presented in Table 2. A total of 10,364 patients were included in the analysis, of whom 1483 (14.3%) developed AKI. The median age and sex distribution were similar in the AKI and non-AKI groups. Of the total patient population, 42.2% had been diagnosed with congestive heart failure, 36.8% with diabetes mellitus, 46.0% with chronic kidney disease, and 56.9% with hypertension. The AKI group showed a significantly higher prevalence of diabetes mellitus (43.2%), chronic kidney disease (56.1%), and hypertension (61.9%). A total of 1581 patients in the total population exhibited severe heart failure symptoms and were categorized as New York Heart Association (NYHA) functional class IV. The AKI group also had a significantly higher percentage of patients categorized as NYHA functional class IV (22.3%) than the non-AKI group (14.1%).

**Table 2:** Baseline characteristics of patients with and without AKI.

Variable	Available number	Total (*n* = 10,364)	AKI (*n* = 1483)	Non-AKI (*n* = 8881)	*P*
**Baseline characteristics**					
Age, years	10,364	75.5 [63.5, 83.0]	74.2 [62.6, 82.2]	75.7 [63.6, 83.1]	0.014
Male	10,364	5683 (54.8)	791 (53.3)	4892 (55.1)	0.211
Previous diagnosis of CHF	10,364	4378 (42.2)	571 (38.5)	3807 (42.9)	0.002
**Underlying diseases**					
Diabetes mellitus	10,364	3819 (36.8)	641 (43.2)	3178 (35.8)	< 0.001
Chronic kidney disease	10,364	4772 (46.0)	832 (56.1)	3940 (44.4)	< 0.001
Hypertension	10,364	5898 (56.9)	918 (61.9)	4980 (56.1)	< 0.001
**Heart function**					
NYHA functional class IV	10,364	1581 (15.3)	330 (22.3)	1251 (14.1)	< 0.001
**LVEF group**	10,364				0.471
< 40% (Reduced)		3617 (34.9)	507 (34.2)	3110 (35.0)	
40–54%		2372 (22.9)	346 (23.3)	2026 (22.8)	
≥ 55% (Preserved)		3996 (38.6)	566 (38.2)	3430 (38.6)	
Unknown		379 (3.7)	64 (4.3)	315 (3.5)	
**Vital signs (first record after admission)**					
SBP, mmHg	10,150	138 [117, 158]	146 [122, 170]	137 [116, 157]	< 0.001
DBP, mmHg	10,151	79 [68, 93]	81 [69, 95]	79 [68, 93]	0.009
Heart rate, beat/min	10,023	88 [74, 104]	91 [76, 106]	88 [74, 104]	< 0.001
**Baseline lab data (first record after admission)**					
Hemoglobin, g/dL	10,364	11.8 [9.9, 13.6]	10.3 [8.7, 12.3]	12.0 [10.2, 13.8]	< 0.001
Platelets, 1000/uL	10,359	196 [151, 251]	195 [148, 250]	196 [152, 251]	0.229
Lymphocyte, %	10,345	17 [11, 25]	14 [9, 21]	17 [11, 25]	< 0.001
BUN, mg/dL	7831	27.0 [18.0, 45.0]	47.0 [27.2, 76.2]	25.1 [17.0, 39.8]	< 0.001
Creatinine, mg/dL	10,364	1.4 [1.0, 2.2]	2.8 [1.4, 5.2]	1.3 [1.0, 1.9]	< 0.001
eGFR, ml/min/1.73^2^	10,364	45.1 [25.9, 66.1]	19.4 [9.6, 44.2]	48.1 [30.2, 68.2]	< 0.001
Bicarbonate, mmol/L	4236	23.5 [19.8, 27.3]	20.7 [17.1, 24.5]	24.1 [20.7, 28.0]	< 0.001
Sodium, mg/dL	10,248	138 [134, 140]	137 [134, 140]	138 [135, 140]	0.001
Potassium, mg/dL	10,360	4.0 [3.6, 4.5]	4.3 [3.7, 4.9]	4.0 [3.6, 4.5]	< 0.001
Albumin, mg/dL	6770	3.4 [3.0, 3.7]	3.3 [2.9, 3.6]	3.4 [3.1, 3.8]	< 0.001
**Proteinuria (U/A dipstick), mg/dL**	6639				< 0.001
Negative (0–4)		2384 (23.0)	188 (12.7)	2196 (24.7)	
Trace (5–29)		698 (6.7)	75 (5.1)	623 (7.0)	
≥ 1 + (≥ 30)		3557 (34.3)	833 (56.2)	2724 (30.7)	
Unknown		3725 (35.9)	387 (26.1)	3338 (37.6)	
BNP, pg/mL	7137	923 [447, 1815]	1226 [603, 2430]	870 [429, 1700]	< 0.001
Lactic acid, mg/dL	1692	17.8 [11.9, 32.5]	20.9 [12.0, 46.0]	17.3 [11.9, 29.0]	< 0.001
pH	3699	7.4 [7.3, 7.5]	7.4 [7.3, 7.4]	7.4 [7.4, 7.5]	< 0.001
**Medication treatment within first 48 h**					
Digoxin	10,364	1031 (9.9)	94 (6.3)	937 (10.6)	< 0.001
Calcium channel blocker	10,364	1937 (18.7)	396 (26.7)	1541 (17.4)	< 0.001
Beta-blocker	10,364	3653 (35.2)	578 (39.0)	3075 (34.6)	0.001
**Loop-diuretics**					
Furosemide dosage, mg/ml	10,364	50 [10, 80]	60 [20, 100]	50 [10, 80]	< 0.001
Out-patient loop diuretics or spironolactone use	10,364	5460 (52.7)	794 (53.5)	4666 (52.5)	0.475

*AKI* acute kidney injury; *BNP* B-type natriuretic peptide; *BUN* blood urea nitrogen; *CHF* congestive heart failure; *DBP* diastolic blood pressure; *LVEF* left ventricular ejection fraction; *NYHA* New York Heart Association; *SBP* systolic blood pressure; *eGFR* estimated glomerular filtration rate.

Data are given as frequency (percentage) or median (25th, 75th percentiles).

*CHF was defined by ICD code recorded in outpatient clinics or previous admission.

^†^CKD was defined by combination of the ICD code in outpatient clinics or previous admission and the eGFR lower than 60 before the index day.

Regarding the clinical parameters, the AKI group patients had significantly higher systemic blood pressure upon admission. The AKI group also exhibited significantly lower haemoglobin, lymphocyte percentage, serum albumin, and bicarbonate levels as well as higher creatinine, blood urea nitrogen, potassium, lactic acid, and BNP levels. A higher percentage of AKI group patients showed positive proteinuria results via dipstick tests. The AKI group received higher dosages of loop diuretics during their AHF admission period; however, there was no significant difference between the groups in outpatient diuretic treatment strategy. The AKI group was more likely to receive calcium channel blockers but less likely to use digoxin during hospitalization (Table 2).

### Validation of existing prediction models for AKI

**Figure 2:**
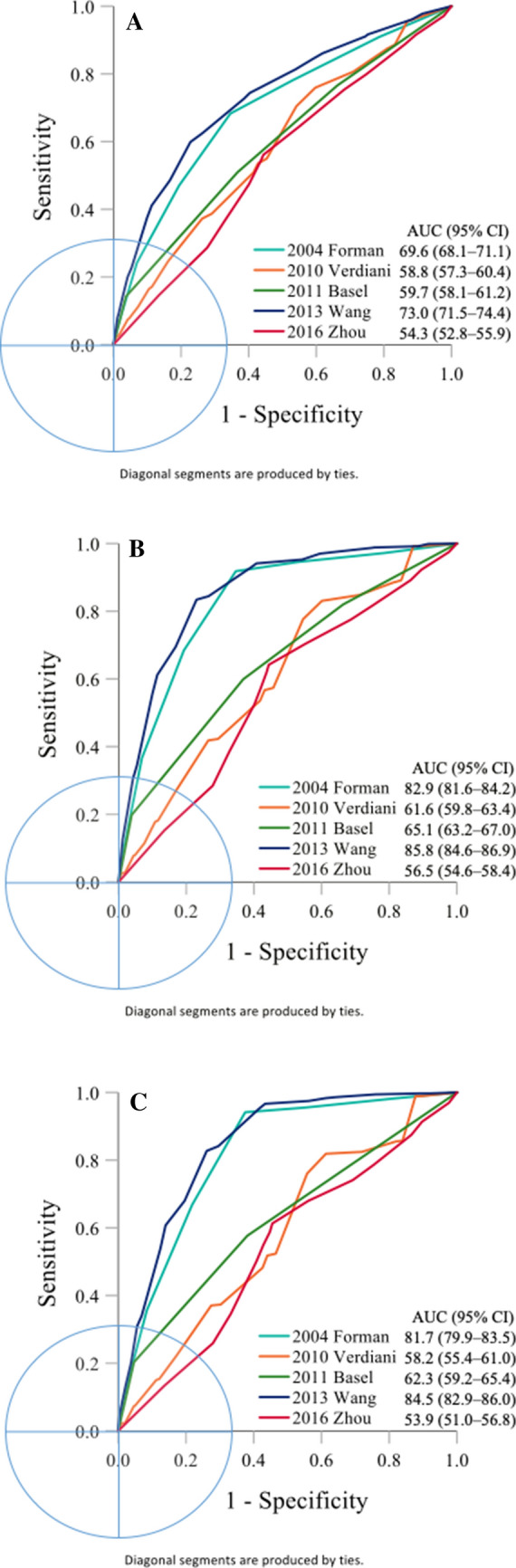
The discrimination ability by assessing the area under the receiver operating characteristic (AUC) curve for AKI (**A**), AKI stage 3 (**B**), and dialysis (**C**). *AKI* acute kidney injury; *CI* confidence interval.

The performance of predicting AKI events in patients with AHF was externally validated for each existing prediction, as summarized in Table 3. The AUC discrimination ability was highest for the Wang et al. model (AUC = 73%), followed by the Forman risk score (69.6%), Basel risk score (59.7%), Verdiani et al. model (58.8%), and Zhou et al. model (54.3%) (Fig. 2A). Regarding calibration, the HL chi-square statistics were the smallest for the Wang et al. model, followed by the Forman risk score, Zhou et al. model, Basel risk score, and Verdiani et al. model (Table 3). The pairwise comparison results for the AUCs showed that all of the AUCs differed significantly between any two prediction models, except for the Basel risk score and Verdiani et al. model (Table 4).

**Table 3:** Prediction model performance in discrimination and calibration outcomes of interest.

Outcome/risk score	AUC (95% CI)^a^	Cutoff^b^	Sensitivity (95% CI)	Specificity (95% CI)	χ^2^ of HL test^c^
**AKI**					
2004 Forman risk score	69.6 (68.1–71.1)	3	68.31 (65.9–70.7)	65.38 (64.4–66.4)	36.2
2010 Verdiani	58.8 (57.3–60.4)	8	70.47 (68.1–72.8)	45.86 (44.8–46.9)	168.4
2011 Basel risk score	59.7 (58.1–61.2)	2	50.98 (48.4–53.6)	63.25 (62.2–64.3)	78.0
2013 Wang	73 (71.5–74.4)	12	59.88 (57.3–62.4)	77.22 (76.3–78.1)	35.4
2016 Zhou	54.3 (52.8–55.9)	10	56.10 (53.5–58.6)	55.64 (54.6–56.7)	68.1
**AKI stage 3**					
2004 Forman risk score	82.9 (81.6–84.2)	3	91.82 (89.8–93.6)	65.34 (64.4–66.3)	81.9
2010 Verdiani	61.6 (59.8–63.4)	8	77.65 (74.7–80.4)	45.46 (44.5–46.5)	328.5
2011 Basel risk score	65.1 (63.2–67.0)	2	59.91 (56.6–63.2)	63.14 (62.2–64.1)	63.9
2013 Wang	85.8 (84.6–86.9)	12	83.41 (80.8–85.8)	76.97 (76.1–77.8)	46.6
2016 Zhou	56.5 (54.6–58.4)	10	64.17 (60.9–67.4)	55.61 (54.6–56.6)	139.3
**Dialysis within 7 days**					
2004 Forman risk score	81.7 (79.9–83.5)	3	94.15 (91.2–96.3)	62.52 (61.6–63.5)	64.3
2010 Verdiani	58.2 (55.4–61.0)	7	81.89 (77.5–85.7)	38.67 (37.7–39.6)	192.3
2011 Basel risk score	62.3 (59.2–65.4)	2	57.66 (52.4–62.8)	61.89 (60.9–62.8)	41.1
2013 Wang	84.5 (82.9–86.0)	12	82.73 (78.4–86.5)	73.87 (73.0–74.7)	40.8
2016 Zhou	53.9 (51.0–56.8)	10	61.28 (56.0–66.3)	54.50 (53.5–55.5)	66.5

*AKI*, acute kidney injury; *AUC*, area under the receiver operating characteristic curve; *CI*, confidence interval; *HL*, Hosmer–Lemeshow.

a: Larger numbers indicate better performance;

b: Determined using the Youdex index;

c: Lower numbers indicate better performance.

**Table 4:** Pairwise comparisons of area under the receiver operating characteristic curve between the prediction models.

Outcome/score	Difference in AUC (95% CI) (Column *vs.* Row) †			
	2004 Forman risk score	2010 Verdiani	2011 Basel risk score	2013 Wang
**AKI**				
2004 Forman risk score	–	–	–	–
2010 Verdiani	10.74 (9.06, 12.42)*	–	–	–
2011 Basel risk score	9.93 (8.29, 11.57)*	− 0.81 (− 2.24, 0.61)	–	–
2013 Wang	− 3.37 (− 4.49, − 2.25)*	− 14.11 (− 15.79, − 12.44)*	− 13.30 (− 15.09, − 11.51)*	–
2016 Zhou	15.25 (13.52, 16.99)*	4.51 (3.46, 5.57)*	5.33 (4.14, 6.52)*	18.63 (16.88, 20.37)*
**AKI stage 3**				
2004 Forman risk score	–	–	–	–
2010 Verdiani	21.28 (19.37, 23.19)*	–	–	–
2011 Basel risk score	17.82 (15.89, 19.75)*	− 3.46 (− 5.19, − 1.74)*	–	–
2013 Wang	− 2.86 (− 3.96, − 1.76)*	− 24.14 (− 26.03, − 22.25)*	− 20.68 (− 22.73, − 18.64)*	–
2016 Zhou	26.38 (24.45, 28.31)*	5.10 (3.84, 6.36)*	8.56 (7.11, 10.02)*	29.25 (27.31, 31.18)*
**Dialysis within 7 days**				
2004 Forman risk score	–	–	–	–
2010 Verdiani	23.52 (20.67, 26.37)*	–	–	–
2011 Basel risk score	19.40 (16.42, 22.38)*	− 4.12 (− 6.69, − 1.54)*	–	–
2013 Wang	− 2.74 (− 4.44, − 1.03)*	− 26.25 (− 29.06, − 23.45)*	− 22.13 (− 25.26, − 19.01)*	–
2016 Zhou	27.83 (24.98, 30.67)*	4.31 (2.39, 6.22)*	8.43 (6.23, 10.62)*	30.56 (27.74, 33.38)*

*Indicates *P* < 0.05;

^†^ DeLong’s test.

### Extension of models for predicting AKI stage 3 and dialysis

Among the 1483 patients with AKI, 519 (35%) were stage 1, 96 (6%) were stage 2, and 868 (58%) were stage 3. Among the 868 patients with stage 3 AKI, 509 (59%) did not undergo dialysis, and 259 (41%) had AKI requiring dialysis. We extended the scores of the prediction models to examine their ability to predict AKI stage 3 and dialysis. The results showed that the Wang et al. study and Forman risk score demonstrated satisfactory discrimination performance (AUC = 85.8% and 82.9%, respectively) for AKI stage 3 and relatively low HL chi-square statistics (Table 3 and Fig. 2B). Similar to the results for predicting AKI stage 3, the discrimination performance of the Wang et al. model (AUC = 84.5%) and Forman risk score (81.7%) for dialysis was satisfactory, and these models had relatively low HL chi-square statistics (Table 3 and Fig. 2C). In addition, all of the AUC pairwise comparisons differed significantly in predicting AKI stage 3 or dialysis.

### Extension of models for predicting MAKEs

**Figure 3:**
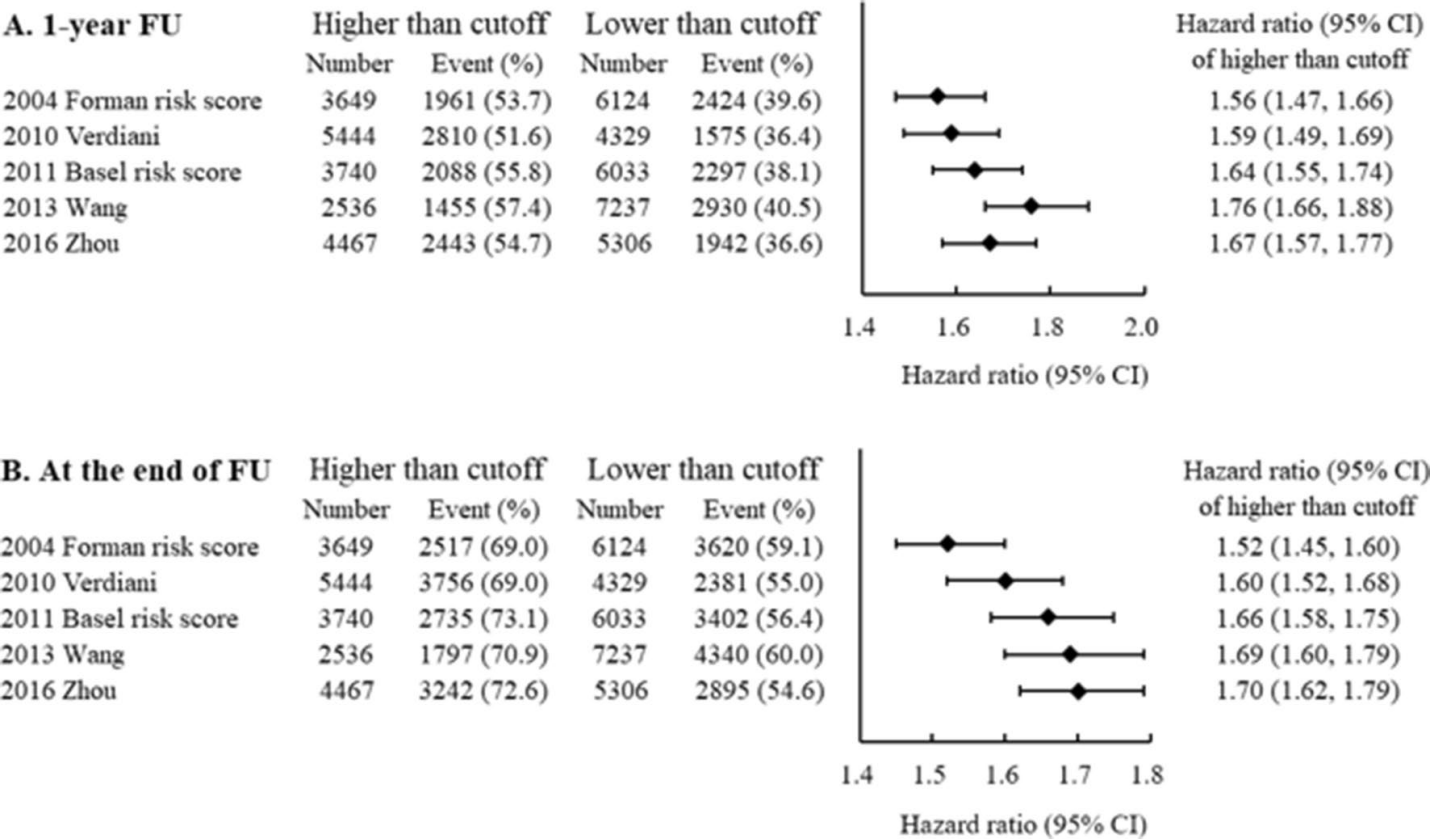
Forest plot showing the association between higher risk scores (above the optimal cutoff) and the risk of MAKEs during 1-year follow-up (**A**) and at the end of follow-up (**B**). *MAKEs* major adverse kidney events.

We next analysed MAKEs within 1 year of AKI diagnosis and MAKEs from the index day to the end of follow-up. A total of 6137 (62.8%) patients suffered from MAKE events, of whom 5437 (55.6%) patients developed CKD, 1298 (13.3%) patients developed ESRD requiring chronic renal replacement therapy, and 2684 (27.5%) died. The patients were separated into two groups according to the cut-off value determined by the Youden index in predicting AKI and AKI stage 3. The results showed that the group with higher risk scores had a significantly greater risk of MAKEs than did the group with lower scores according to all five models, of which the hazard ratios ranged from 1.56 to 1.76 for 1-year follow-up and 1.52–1.70 for the index date to the end of follow-up (Fig. 3A,B).

### Additional analysis

We further conducted an additional analysis by excluding the patients with a creatinine level > 3.5 at arrival of the index admission because those patients may not be right at the cusp of developing severe AKI and may be lower on the nonlinear creatinine curve. The analyses demonstrated similar results to the overall results in that the performance of discrimination and calibration of the Wang et al. study and Forman risk score was superior to that of the other three scores (Supplemental Table 1). In addition, the results were generally consistent with the overall results when using the complete data set without any missing values (Supplemental Table 2).

## Discussion

In the present study, we externally validated five existing models for predicting the risk of AKI in patients with AHF. The Forman risk score and Wang et al. model showed superior discrimination and calibration performance compared with the three other models.

The development of AKI in patients with AHF leads to prolonged hospitalization, increased readmission rates, and increased short- and long-term all-cause mortality and cardiovascular mortality. Coexisting AKI and AHF also lead to higher health care costs for patients with heart failure^4–6^. In the past two decades, many studies have focused on the early identification of patients with AHF who are at high risk of AKI development to initiate intervention earlier and improve their clinical outcomes. Some of these studies have used clinical parameters as risk predictors, and others have introduced or added novel urine biomarkers for AKI prediction^1,13–16^. However, the widely varying definition and classification of AKI (or WRF in some studies) as well as differences in the observed time-at-risk and heterogeneity of study populations have hindered the cross-comparison of published data. For this reason, AKI in the present study was defined according to the KDIGO Clinical Practice Guidelines for Acute Kidney Injury published in 2012^20^, which are currently the most widely accepted and used criteria. To our knowledge, this is the first multi-institution validation study to use the KDIGO guidelines to compare existing prediction models of AKI in patients with AHF.

Among the AKI prediction models for patients with AHF, the Forman risk score, which was the first to be published, utilizes 4 factors (i.e., congestive heart failure history, diabetes mellitus, systolic blood pressure over 160 mmHg during admission, and elevated creatinine). The study introducing the risk score showed predictive ability for AKI in AHF, but it did not report any area under the ROC curve^13^. The AUC for AKI prediction was externally validated as being 0.65 by Breidthardt et al. in 2011^1^ and Wang et al. in 2013^15^. The subsequently developed Basel risk score sought to use fewer predictive factors to achieve better prediction ability. Chronic kidney disease, bicarbonate level, and outpatient diuretic treatment were used for AKI prediction, and the AUC was reported to be 0.71 in the original article. However, a few years later, Wang et al. found no difference in discrimination ability between the Basel and Forman risk scores, both of which had an AUC of 0.65 according to externally validated results^15^. In 2013, Wang et al. reported a prediction score derived from a larger patient number and, for the first time, included proteinuria as one of the risk factors for AKI prediction in the AHF population. Since then, proteinuria has been increasingly reported to be not only a predictive factor but also an aggravating factor in AKI^25–27^. The Wang et al. prediction model had a high sensitivity of 70.0%, specificity of 70.6%, and AUC of 0.76 in predicting AKI in AHF patients. Subsequently, Zhou et al. derived the first scoring system combining clinical risk factors and novel kidney injury biomarkers (uNGAL and uAGT)^16^. Zhou et al. reported the AUC separately; the AUC for the clinical model alone was 0.765, close to that of the Wang et al. model, while the AUC for the prediction model was 0.874^16^. These five AKI prediction models each have their own advantages and disadvantages in clinical application. The Forman risk score uses only four factors, and each of them is easily and widely examined in the clinical practice. Similar to the Basel risk score, which only included three prediction factors, it was easy for clinicians to use. Wang et al. and Zhou et al. published prediction models and included laboratory parameters that had been reported as AKI aggravating factors or novel AKI biomarkers. Although these markers might provide more information in AKI prediction, they have not been widely examined and are more expensive to assess. This means that using these prediction models was more costly. To offer an easier and more cost-effective choice, we externally validated these five prediction models in the present study.

Our current study not only externally validated these five prediction models in terms of AKI prediction but also estimated their performance in predicting serious AKI events, including AKI stage 3 and dialysis. As Table 3 shows, the AUCs of these prediction models for AKI prediction ranged from 0.543 to 0.73. Better performance was noted in AKI stage 3 and dialysis prediction, with AUCs of 0.565–0.858 and 0.539–0.845, respectively. All five prediction models showed favourable ability in long-term outcome prediction, with significantly higher incidences of MAKEs in the high-score groups than in the low-score groups. Thus, these prediction models can not only predict AKI events in AHF patients during hospitalization but also predict long-term adverse events in AHF patients.

Of the five prediction models we validated, the Forman risk score and Wang et al. model showed superior discrimination and calibration. The AKI risk score for AHF derived using the Wang et al. model had the best performance; its AUC was 0.73 in AKI prediction, and its AUCs for AKI stage 3 and dialysis were 0.858 and 0.845, respectively. This scoring system showed favourable calibration in predicting all three outcomes. The Forman risk score also showed good performance and calibration in AKI, AKI stage 3, and dialysis prediction, with AUCs of 0.696, 0.829, and 0.817, respectively.

Although the further pairwise comparison of AUCs revealed significant differences between the Wang et al. model and Forman risk score (Table 4), both had excellent discrimination (AUC of 0.8–0.9) by general definition^28^. Considering this, the Forman risk score may be seen as a relatively easier and more convenient tool for predicting AKI in AHF patients clinically because it requires only 4 clinical factors.

Much current research is being conducted to identify serum or urine biomarkers for early AKI prediction. However, these biomarkers are more costly to utilize and have not yet been widely examined in general laboratory settings. Some recent studies have reported that adding urine biomarkers to clinical prediction models yielded no significant performance improvement^29–33^, and Törnblom et al. even reported that new statistical methods no longer support using uNGAL to predict AKI in certain patient groups^32^. Taking this into consideration, prediction models based on clinical parameters seem to offer a faster, cheaper, and easier means of AKI prediction, thus increasing the likelihood of AKI prevention and early intervention. The current study demonstrated that a clinical prediction model alone can provide excellent discrimination ability for AKI in AHF patients. Clinical prediction models can achieve an AUC of 0.80, which is particularly high for serious AKI event prediction.

### Strengths and limitations

Our study has several notable strengths. First, this is the first multi-institution validation study to compare existing prediction models of AKI in AHF patients based on the KDIGO Clinical Practice Guidelines. Second, our study further evaluated the performance of these prediction models in predicting serious AKI events and revealed that these prediction models also offer high discriminative power for predicting AKI stage 3 and dialysis. Third, this study not only assessed the short-term renal outcomes of patients with AHF but also evaluated their long-term outcomes. We demonstrated that patients with scores above the cut-off value had poorer long-term outcomes (defined by MAKE incidence) than did patients with lower scores.

This study also has some limitations. First, this was a retrospective analysis, and the inherent drawbacks of this design cannot be avoided. Second, the first record of the creatinine level upon emergency department admission was used as the baseline creatinine level, and AKI was defined by the subsequent change in creatinine. Thus, our study could only examine predictive ability in terms of AKI development during admission and not AKI at admission. For patients with higher baseline creatinine levels, small changes in eGFR could lead to a 0.3 mg/dL increase according to the KDIGO guideline definition. Third, data limitations prevented some prediction factors from being validated, including NT-proBNP, uNGAL, and uAGT. Last, the present study was based on CGRD data, so the enrolled patients were relatively homogenous. The result of external validation in this study might not be applicable to other populations.

## Conclusion

We externally validated five existing prediction models for AKI in patients with AHF. The Forman risk score and Wang et al. model showed favourable discrimination and calibration in predicting AKI, AKI stage 3, and dialysis. The Forman risk score, as it comprises only 4 prediction factors, may offer the easiest and fastest means of individual risk prediction as well as risk stratification. By utilizing appropriate prediction models, clinicians can assess the risk of AKI in patients with AHF earlier and thus plan and initiate adequate disease management for these patients in a much timelier manner.

## Supplementary Information

Supplementary Information.

## Abbreviations

AKI: Acute kidney injury
AHF: Acute heart failure
AUC: Area under the receiver operating characteristic curve
CGRD: Chang Gung Research Database
KDIGO: Kidney disease improving global outcomes
MAKEs: Major adverse kidney events
WRF: Worsening renal function

## References

[1] Breidthardt T (2011). Effect and clinical prediction of worsening renal function in acute decompensated heart failure. Am. J. Cardiol..

[2] Bagshaw SM (2010). Epidemiology of cardio-renal syndromes: workgroup statements from the 7th ADQI Consensus Conference. Nephrol. Dial. Transplant..

[3] Rastogi A, Fonarow GC (2008). The cardiorenal connection in heart failure. Curr. Cardiol. Rep..

[4] Cowie MR (2006). Prevalence and impact of worsening renal function in patients hospitalized with decompensated heart failure: results of the prospective outcomes study in heart failure (POSH). Eur. Heart J..

[5] Chen YT (2020). Acute kidney disease and acute kidney injury biomarkers in coronary care unit patients. BMC Nephrol..

[6] Logeart D (2008). Transient worsening of renal function during hospitalization for acute heart failure alters outcome. Int. J. Cardiol..

[7] Zhou Q (2012). Acute and acute-on-chronic kidney injury of patients with decompensated heart failure: impact on outcomes. BMC Nephrol..

[8] Smith GL (2003). Worsening renal function: what is a clinically meaningful change in creatinine during hospitalization with heart failure?. J. Card. Fail..

[9] Afsar B (2016). Focus on renal congestion in heart failure. Clin. Kidney J..

[10] Fudim M (2018). Worsening renal function during decongestion among patients hospitalized for heart failure: findings from the evaluation study of congestive heart failure and pulmonary artery catheterization effectiveness (ESCAPE) trial. Am. Heart J..

[11] Metra M (2018). Prognostic significance of creatinine increases during an acute heart failure admission in patients with and without residual congestion: a post hoc analysis of the PROTECT data. Circ. Heart Fail..

[12] Mullens W (2019). The use of diuretics in heart failure with congestion—a position statement from the Heart Failure Association of the European Society of Cardiology. Eur. J. Heart Fail..

[13] Forman DE (2004). Incidence, predictors at admission, and impact of worsening renal function among patients hospitalized with heart failure. J. Am. Coll. Cardiol..

[14] Verdiani V, Lastrucci V, Nozzoli C (2010). Worsening renal function in patients hospitalized with acute heart failure: risk factors and prognostic significances. Int. J. Nephrol..

[15] Wang YN, Cheng H, Yue T, Chen YP (2013). Derivation and validation of a prediction score for acute kidney injury in patients hospitalized with acute heart failure in a Chinese cohort. Nephrology (Carlton).

[16] Zhou LZ (2016). Development and validation of a risk score for prediction of acute kidney injury in patients with acute decompensated heart failure: a prospective cohort study in China. J. Am. Heart Assoc..

[17] Alattar FT, Imran N, Debari VA, Mallah KN, Shamoon FE (2010). Fractional excretion of sodium predicts worsening renal function in acute decompensated heart failure. Exp. Clin. Cardiol..

[18] Bellomo R (2004). Acute renal failure—definition, outcome measures, animal models, fluid therapy and information technology needs: the second international consensus conference of the acute dialysis quality initiative (ADQI) group. Crit Care.

[19] Mehta RL (2007). Acute kidney injury network: report of an initiative to improve outcomes in acute kidney injury. Crit Care.

[20] Section 2: KI Definition. *Kidney Int Suppl (2011)* **2**, 19–36. Doi:10.1038/kisup.2011.32 (2012). 10.1038/kisup.2011.32 PMC4089595 25018918

[21] Tsai MS (2017). Chang Gung research database: a multi-institutional database consisting of original medical records. Biomed. J..

[22] Shao SC (2019). The Chang Gung research database-a multi-institutional electronic medical records database for real-world epidemiological studies in Taiwan. Pharmacoepidemiol. Drug Saf..

[23] Billings FTT, Shaw AD (2014). Clinical trial endpoints in acute kidney injury. Nephron Clin Pract.

[24] Palevsky PM (2018). Endpoints for clinical trials of acute kidney injury. Nephron.

[25] Chen J-J (2017). Proteinuria enhances prediction ability of sequential organ failure assessment score and associated with mortality in coronary care units. Acta Nephrologica.

[26] Hsu CY (2020). Post-acute kidney injury proteinuria and subsequent kidney disease progression: the assessment, serial evaluation, and subsequent sequelae in acute kidney injury (ASSESS-AKI) study. JAMA Intern. Med..

[27] Huang TM (2011). Preoperative proteinuria predicts adverse renal outcomes after coronary artery bypass grafting. J. Am. Soc. Nephrol..

[28] Mandrekar JN (2010). Receiver operating characteristic curve in diagnostic test assessment. Biostat. Clin..

[29] Alobaidi R, Basu RK, Goldstein SL, Bagshaw SM (2015). Sepsis-associated acute kidney injury. Semin. Nephrol..

[30] Flechet M (2017). AKIpredictor, an online prognostic calculator for acute kidney injury in adult critically ill patients: development, validation and comparison to serum neutrophil gelatinase-associated lipocalin. Intensive Care Med..

[31] Ronco C (2014). Neutrophil gelatinase-associated lipocalin: ready for routine clinical use? An international perspective. Blood Purif..

[32] Törnblom S (2020). Urine NGAL as a biomarker for septic AKI: a critical appraisal of clinical utility—data from the observational FINNAKI study. Ann. Intensive Care.

[33] Albert C (2018). Urinary neutrophil gelatinase-associated lipocalin-guided risk assessment for major adverse kidney events after open-heart surgery. Biomark. Med..

