# Supplementary material for: A validation study comparing existing prediction models of acute kidney injury in patients with acute heart failure
Source: Sci Rep. 2021 May 27;11:11213. doi: 10.1038/s41598-021-90756-9 (PMC8159983; doi:10.1038/s41598-021-90756-9)
Supplement: Supplementary file 1 — Supplementary Information. [file 41598_2021_90756_MOESM1_ESM.docx]

**Supplemental Table 1**. Prediction model performance in discrimination and calibration outcomes of interest, excluding the patients whose creatinine level >3.5 at arrival of the index admission (*n* = 9,186)

| Outcome/risk score | AUC (95% CI)^a^ | Cutoff^b^ | Sensitivity (95% CI) | Specificity (95% CI) | χ^2^ of HL test^c^ |
| --- | --- | --- | --- | --- | --- |
| AKI |  |  |  |  |  |
| 2004 Forman risk score | 59.2 (58.2 – 60.2) | 3 | 45.85 (42.5 - 49.2) | 69.80 (68.8 - 70.8) | 7.8 |
| 2010 Verdiani | 57.9 (56.8 – 58.9) | 7 | 70.51 (67.3 - 73.5) | 42.23 (41.2 - 43.3) | 21.6 |
| 2011 Basel risk score | 56.4 (55.4 – 57.4) | 2 | 43.43 (40.1 - 46.8) | 65.03 (64.0 - 66.1) | 2.3 |
| 2013 Wang | 63.6 (65.6 – 64.6) | 9 | 56.22 (52.8 - 59.6) | 63.69 (62.6 - 64.7) | 7.8 |
| 2016 Zhou | 53.8 (52.8 – 54.8) | 6 | 74.31 (71.3 - 77.2) | 32.75 (31.7 - 33.8) | 9.2 |
| AKI stage 3 |  |  |  |  |  |
| 2004 Forman risk score | 72.8 (71.9 – 73.7) | 3 | 71.94 (66.0 - 77.4) | 69.46 (68.5 - 70.4) | 17.5 |
| 2010 Verdiani | 63.5 (62.5 – 64.5) | 7 | 81.82 (76.5 - 86.4) | 41.68 (40.7 - 42.7) | 44.5 |
| 2011 Basel risk score | 64.7 (63.7 – 65.7) | 2 | 55.73 (49.4 - 62.0) | 64.79 (63.8 - 65.8) | 3.1 |
| 2013 Wang | 77.7 (76.9 – 78.6) | 12 | 62.06 (55.8 - 68.1) | 81.37 (80.5 - 82.2) | 19.8 |
| 2016 Zhou | 57.8 (56.8 – 58.9) | 8 | 58.10 (51.8 - 64.3) | 57.39 (56.4 - 58.4) | 7.6 |
| Dialysis within 7 days |  |  |  |  |  |
| 2004 Forman risk score | 71.2 (70.3 – 72.1) | 3 | 71.23 (59.4 - 81.2) | 68.64 (67.7 - 69.6) | 6.6 |
| 2010 Verdiani | 60.6 (59.6 – 61.6) | 7 | 82.19 (71.5 - 90.2) | 41.22 (40.2 - 42.2) | 25.4 |
| 2011 Basel risk score | 61.1 (60.1 – 62.1) | 1 | 82.19 (71.5 - 90.2) | 34.37 (33.4 - 35.4) | 1.4 |
| 2013 Wang | 77.4 (76.5 – 78.2) | 9 | 83.56 (73.0 - 91.2) | 62.17 (61.2 - 63.2) | 12.0 |
| 2016 Zhou | 53.7 (52.7 – 54.7) | 8 | 52.05 (40.0 - 63.9) | 57.04 (56.0 - 58.1) | 4.4 |

AKI, acute kidney injury; AUC, area under the receiver operating characteristic curve; CI, confidence interval; HL, Hosmer–Lemeshow.

a: Larger numbers indicate better performance;

b: Determined using the Youdex index;

c: Lower numbers indicate better performance.

**Supplemental Table 2**. Discrimination performance of interested outcomes using the complete data set (*n* = 3,080)

| Outcome/risk score | AUC, % | 95% CI of AUC | χ^2^ of HL test^a^ |
| --- | --- | --- | --- |
| AKI |  |  |  |
| 2004 Forman risk score | 66.1 | 63.9 - 68.3 | 13.9 |
| 2010 Verdiani | 56.8 | 54.5 - 59.2 | 33.5 |
| 2011 Basel risk score | 59.2 | 56.9 - 61.5 | 11.4 |
| 2013 Wang | 68.1 | 65.8 - 70.3 | 15.8 |
| 2016 Zhou | 53.0 | 50.7 - 55.4 | 27.6 |
| AKI stage 3 |  |  |  |
| 2004 Forman risk score | 79.1 | 77.1 - 81.2 | 39.0 |
| 2010 Verdiani | 59.6 | 56.9 - 62.2 | 76.1 |
| 2011 Basel risk score | 65.2 | 62.6 - 67.8 | 9.3 |
| 2013 Wang | 80.6 | 78.6 - 82.6 | 15.3 |
| 2016 Zhou | 55.5 | 52.8 - 58.3 | 54.3 |
| Dialysis within 7 days |  |  |  |
| 2004 Forman risk score | 77.7 | 74.8 - 80.6 | 26.4 |
| 2010 Verdiani | 57.4 | 53.5 - 61.3 | 42.4 |
| 2011 Basel risk score | 62.7 | 58.7 - 66.7 | 5.1 |
| 2013 Wang | 77.8 | 75.0 - 80.6 | 17.7 |
| 2016 Zhou | 52.7 | 48.6 - 56.8 | 39.5 |

AKI, acute kidney injury; AUC, area under the receiver operating characteristic curve; CI, confidence interval;

a: Lower numbers indicate better performance.
